# A Brief History of COPD: As Told by Some of Its Senior Scientists and Clinicians

**DOI:** 10.3390/jcm15103914

**Published:** 2026-05-19

**Authors:** Linda Nici, Bartolome R. Celli, David Mannino, Steve I. Rennard, Alvar Agusti, Suzanne Lareau, Paula Meek, Denis O’Donnell, J Alberto Neder, Jadwiga A. Wedzicha, Richard Casaburi, Roger Goldstein, Carolyn L. Rochester

**Affiliations:** 1 The Warren Alpert Medical School, Brown University, 222 Richmond St., Providence, RI 02903, USA; 2 Department of Medicine, Harvard Medical School, Boston, MA 02115, USA; 3 COPD Foundation, Miami, FL 33134, USA; 4 Department of Medicine, College of Medicine, University of Kentucky, Lexington, KY 40511, USA; 5 Department of Internal Medicine, University of Nebraska Medical Center, Omaha, NE 68198, USA; 6 Respiratory Institute, Hospital Clinic, University of Barcelona, 08007 Barcelona, Spain; aagusti@clinic.cat; 7 College of Nursing, University of Colorado, Denver Anschutz Medical Campus, ED 2 North, 13120 East 19th Ave., Aurora, CO 80045, USA; 8 College of Nursing, University of Utah, 10 2000 E, Salt Lake City, UT 84112, USA; 9 Department of Medicine, Queen’s University, Kingston, ON K7L 3N6, Canada; 10 National Heart and Lung Institute, Imperial College London, London SW7 2AZ, UK; 11 Division of Respiratory and Critical Care Physiology and Medicine, Respiratory Research Center, Lundquist Institute at Harbor-UCLA Medical Center, Torrance, CA 90502, USA; casaburi@ucla.edu; 12 Departments of Respiratory Medicine and Physical Therapy, University of Toronto, West Park Healthcare Centre (UHN), Toronto, ON M6M 2J5, Canada; 13 Section of Pulmonary, Critical Care and Sleep Medicine, Yale University School of Medicine, New Haven, CT 06520, USA

**Keywords:** COPD, emphysema, chronic bronchitis, nomenclature, natural history, patient-reported outcomes, PRO, hyperinflation, oxygen, exacerbations, pulmonary rehabilitation

## Abstract

Chronic obstructive pulmonary disease (COPD), which includes chronic bronchitis and emphysema, is highly prevalent worldwide and is the third leading cause of death. While some aspects of the disease were known since the Enlightenment, Laennec’s work in the 19th century began the process of our current understanding of this disease. In this narrative review, 13 clinicians and scientists with over three centuries of cumulative experience treating and studying COPD give their perspectives on the science underpinning our modern concept of this disease and its management. These include (1) the challenges of coming up with a name for what is a complex syndrome; (2) the evolution of our thinking on the natural history of the disease; (3) the importance of particulate matter inhalation in its pathogenesis; (4) the often-overlooked but important—and often treatable—systemic effects of the disease that contribute to its morbidity and mortality; (5) the changes in our perspective of not just addressing pathologic or physiologic abnormalities but also measuring outcomes, such as breathlessness or health-related quality of life, that are of considerable importance to the patient; (6) the role of pharmacologic therapy in not only providing symptomatic relief by increasing airway caliber but also in disease modification, especially by reducing exacerbation frequency; (7) lung hyperinflation as an essential feature of COPD pathophysiology, driving symptom burden, exercise limitation, and mortality risk; (8) long-term oxygen therapy, despite being demonstrated to prolong survival in a defined set of hypoxemic patients with COPD, still having unanswered questions regarding its application and delivery; and (9) pulmonary rehabilitation, a major component of the non-pharmacologic treatment of COPD patients and prominently situated in clinical guidelines for this disease. While this, by necessity, must be a brief review of a very complex disease, the perspectives of these esteemed clinicians and scientists should be of use to other clinicians in understanding and managing this disease.

## 1. Introduction

Chronic obstructive pulmonary disease (COPD) affects more than 400 million people worldwide, leading to significant socioeconomic and health consequences. Despite advances in our understanding and management of this disease, it remains a global health challenge, ranking as the third leading cause of death [1]. COPD is defined by the 2025 GOLD report as a “*heterogeneous lung condition characterized by respiratory symptoms such as dyspnea*, *cough*, *and sputum production. These symptoms result from airway (bronchitis*, *bronchiolitis) and/or alveolar (emphysema) abnormalities*, *leading to persistent*, *often progressive*, *airflow limitation. COPD is associated with an enhanced chronic inflammatory response in the airways and lungs to noxious particles or gases. Exacerbations and comorbidities significantly contribute to the overall severity in individual patients*” [2].

Historically, COPD was seen as a disease predominantly caused by tobacco smoking. This view has been challenged in recent years as accumulating evidence highlights the significant role of other risk factors, including pollution; occupational exposure; and early life insults, including premature birth and childhood respiratory infections. Additionally, the heterogeneity of COPD has become increasingly evident, with phenotypes and endotypes defined by distinct clinical, physiological, and biological characteristics [3].

The clinical trajectory of COPD also varies widely among patients. Some individuals experience a slow decline in lung function, while others progress rapidly or experience frequent exacerbations that accelerate disease progression. These differences emphasize the need for a personalized approach to COPD management that integrates genetic, environmental, and clinical data to optimize outcomes.

In this brief narrative review, expert clinicians and scientists with over three centuries of cumulative experience in the field had structured discussions as to key themes that would best reflect our growing recognition of the complexity of COPD. The results of these discussions are now presented in this review, which explores the evolving nature of our understanding of COPD, from its definition and natural history, pathobiology, and pathophysiology to its systemic manifestations and comorbidities. We explore the ever-broadening complexities of this disease and stress the need for a multidimensional approach to treatment that considers the outcomes of most importance to the patient.

## 2. What’s in a Name? Our Muddled History of Defining COPD

It was Aristotle who first proposed that a definition should state the essential nature or being of something. However, this abstract concept does not translate well to medical practice, where precise definitions are needed to communicate with colleagues and patients and to conduct epidemiological, clinical, translational, and discovery research in the name of scientific progress. There are existing basic rules to name or define a subject, an object, or a disease [4]. Thus, a definition must describe its essential attributes while avoiding circularity; it must also not consist of terms that are synonymous with it. It should not be too wide or too narrow (not miss or include anything to which the term should not be applied). It should also be clear, understandable, and positive, attempting to avoid concepts derived by exclusions. If a definition is too complex, there is also a risk of losing or diminishing its effectiveness in exchange for greater explanatory power. It is also important to highlight that definitions in medicine and other empirical sciences are not rigid and are designed to evolve over time. With these general concepts about what a definition should do, we can then travel over time to observe how the COPD name came to be.

### 2.1. The Beginning

While performing autopsies of persons dying from different diseases, the Swiss pathologist Theophile Bonet in the 1600s and the Italian anatomist Giovanni Morgagni in the 1700s observed that the lungs of some of those persons were “voluminous” or “turgent”, a fact that to them was suggestive of air-trapping [5]. However, they did not relate their findings to any clinical manifestation of disease. It took the inquisitive mind of René Laennec in the year 1816 A.D. to empirically relate the presence of pathological changes in the lung of a person dying from what the British physician Charles Badham had described earlier that century as suffering from “catarrh” to a more precise definition that reflected the nature of the disease itself [6]. He called this disease “emphysema” from the Greek “to inflate”. Not only did he provide a name for this condition, but he also postulated that the cause of this trapping of air had as its root the destruction of the lung parenchyma. Laennec even wrote: “*In opening the chest*, *it is not unusual to find that the lungs do not collapse*, *but they fill up the cavity completely on each side of the heart. When experienced*, *this will appear full of air. The bronchus of the trachea is often at the same time filled with mucous fluid*” [7]. Thus, Laennec had already described the frequent association between emphysema and bronchitis. This was the first nosological description and naming of a specific condition that differed from other respiratory conditions that cause similar symptoms but did not have the characteristic anatomo-pathological features of emphysema.

### 2.2. From Pathologic Anatomy to Physiological Dysfunction

Another giant step was taken when John Hutchinson, an English surgeon, built the first spirometer in 1846 and, for the first time, offered the possibility of measuring the different lung volumes and capacities [8]. Although Hutchinson had already labeled the maximal volume of air that a person could inhale and then exhale as the “vital capacity”, it was not until 1947 when Tiffeneau and Pinelli introduced the concept of timed vital capacity, providing the physiological basis for the diagnosis of diseases that could limit the flow of air during a forced exhalatory maneuver [9]. Finally, a clinical pathological condition could be explored using a test of the function of the organ system in an individual.

### 2.3. Organizing the Acquired Knowledge

By the late 1950s, the pulmonary community felt it was necessary to precisely define the spectrum of respiratory pathologies that were being diagnosed more frequently in patients, particularly adults with a history of smoking. First, in the CIBA symposium [10], then by the American Thoracic Society [11], the definitions of chronic bronchitis and emphysema were finally accepted as related respiratory diseases. Thus, the term chronic bronchitis was defined as a productive cough that lasts at least 3 months over 2 consecutive years, while emphysema continued to be defined pathologically as enlargement of the alveoli in the lungs due to destruction of normal parenchymal structure. The subsequent years cemented an association between exposure to toxic agents, and the frequent coexistence of bronchitis and emphysema in single patients.

### 2.4. The Name Was Born

Integrating the scientific advances that had occurred since Laennec’s description, in 1965, at the ninth Aspen lung conference, Dr. William Briscoe is credited with using the term chronic obstructive pulmonary diseases (COPDs) to define a group of diseases that were characterized by spirometrically defined obstruction to expiratory airflow, usually associated with chronic bronchitis and suspected emphysema [12]. The name became well accepted in the medical community [13], although much less so in the general population, where, despite its importance as a cause of disability and death, it remains unrecognized by large segments of the population. In 1976, Fletcher and co-workers published a landmark study that centered on the accelerated decline in lung function in current smokers in a cohort of train workers in London, which is the nature of COPD [14]. With the worldwide increase in cigarette smoking around the world, this disease became a major public health problem around the world, and the modern era of COPD research was born. It is interesting that Dr. Briscoe had a letter “s” at the end of the disease, to imply that these were diseases, a concept that may gain relevance today, as we now know that there are many causative agents of airflow obstruction.

### 2.5. Moving Upstream with Imaging and “Omics”

Recent decades have witnessed an explosion of information around the different causes of COPD, its many phenotypes and endotypes, and its diverse clinical expressions and progression [14,15,16,17,18]. This has been in part the natural consequence of incredible advances in imaging and the widespread use of high-throughput “omics” [19], which have exponentially expanded our knowledge in many different medical fields, including that of COPD. To begin, images obtained with computed chest tomography can now diagnose with accuracy the degree and distribution of emphysema, as well as the quantification of the airways, their thickness, and the presence or absence of intraluminal mucous (14). In addition, it has been shown that there are persons with non-obstructive spirometry who complain of symptoms like cough and sputum production, others who have abnormal decline in lung function, or others in whom early imaging features indicate the presence of emphysema and who are at risk of developing clinical COPD in a relatively short period of time. The need to name this “early” phase of the COPD continuum has given rise to the term of pre-COPD [20]. Raising the hope that instead of waiting for these persons to develop advanced disease, we shall, in the not-too-distant future, prevent or reverse disease progression with novel and yet to be developed therapies. Also, given that there are different causes of chronic airflow obstruction, perhaps we should consider going back to the concept of COPDs [20].

**Figure 1:**
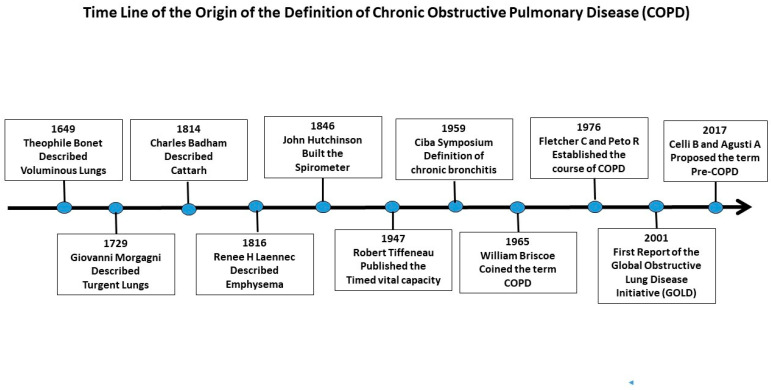
Timeline of the origin of the definition of chronic obstructive pulmonary disease (COPD). Timeline of the evolution of the naming of the disease we now recognize as chronic obstructive pulmonary disease.

These advances are improving our capacity to more precisely define the different aspects of COPD. As has already occurred with other respiratory diseases, such as tuberculosis, once defined as phthisis or consumption, the accumulation of knowledge in the field of COPD may lead to changes in its definition to better serve our peers and patients. A brief outline of our evolving concept of COPD is given in Figure 1.

## 3. The Natural History of COPD over the Individual’s Lifespan

**Figure 2:**
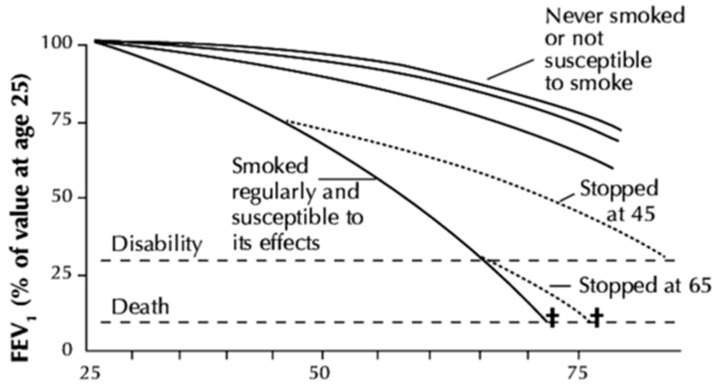
Lung function decline as depicted by Fletcher and Peto in their 1976 publication [14].

In their landmark work, Fletcher and Peto described the natural history of lung function decline based on their study of 792 men aged 30–59 years old followed for 8 years [14,21]. In their classic figure of loss of lung function in subsets of the population (susceptible smokers, non-susceptible smokers, and susceptible smokers who quit smoking at different ages), they depicted forced expiratory volume in 1 s (FEV_1_) decline relative to age 25 (Figure 2) [14].

Some features of this depiction are that never smokers do not have accelerated loss of lung function, people with accelerated loss of lung function who stop smoking will revert to a less steep decline, and the rate of lung function decline increases over time (the “horse racing effect”). In the nearly fifty years since the publication of this seminal work, our knowledge of COPD, its risk factors, and natural history has expanded dramatically, and many of these features are now challenged.

**Figure 3:**
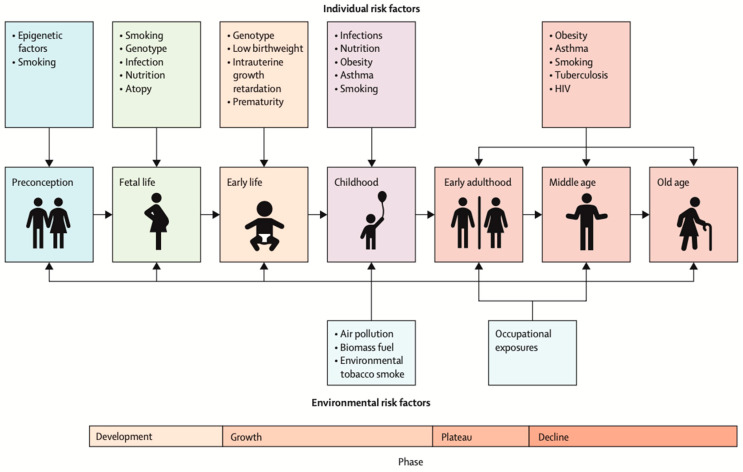
Individual and environmental risk factors for the development and progression of chronic obstructive pulmonary disease. Reproduced with permission from reference [22].

The origins of COPD are now known to be earlier than in adulthood, when the disease is typically diagnosed. Figure 3 demonstrates the individual and environmental risk factors that go back to the preconception period [22].

**Figure 4:**
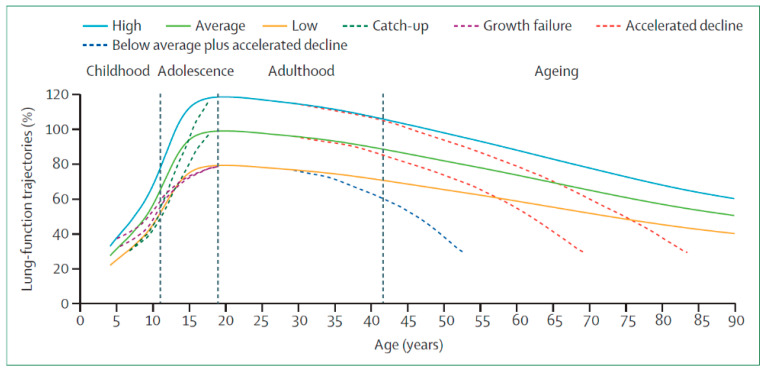
Lung-function trajectories from childhood to adulthood (the trajectome). For further explanations, see the text. Reproduced with permission from reference [26].

COPD is the result of a number of gene (G)–environment (E) interactions that can occur over the lifetime (T) of the individual (GETomics) that eventually modify the normal lung function trajectory [3] (green trajectory in Figure 4), which is characterized by a *growth phase* during infancy and adolescence that reaches a peak at about 25 years of age (earlier in females [23]) and is followed by a slow decline in lung function with age due to physiological lung aging [24]. Of note, recent research has shown that, contrary to what was previously believed, there is no *plateau* phase after the peak [25]. It is now recognized that lung function trajectory can be altered, both in the growing and/or declining phases (*trajectome*) (Figure 4) [26].

Lung growth can be jeopardized by many factors, including prematurity or small for gestational age at birth, exposure to tobacco or indoor biomass combustion, repeated pulmonary infections, or malnutrition, among other factors [24]. Between 4 and 12% of young adults in the general population have suboptimal peak lung function [24]. This is clinically relevant because these individuals also have a higher prevalence and earlier incidence of cardiovascular and metabolic disorders; die prematurely [27]; and, contrary to what was postulated by Fletcher and Peto [14], may develop COPD without accelerated decline of lung function [16]. Two other observations are relevant in this context. First, for still unclear reasons, some children can “*catch-up*” during infancy and regain a normal lung function trajectory. It is possible that if the original cause of having poor lung function in childhood is environmental, reducing such exposure can contribute to catch-up [28,29]. Second, about 5% of young adults have “supra-normal” peak lung function [30]. These individuals also show healthier aging, with reduced incidence of hospitalizations and comorbidities [31]. This is why spirometry should be viewed not only as a diagnostic tool for respiratory disease but also as a global health marker of both pulmonary and extrapulmonary diseases across the lifespan [32]. It has been recently proposed that spirometry (or other surrogate lung function measurements like the forced oscillation technique (FOT)) should be monitored through life, starting in infancy [33]. A “*lung tracker*” tool has been developed for that purpose and is freely downloadable from the website of the European Respiratory Society (https://gli-calculator.ersnet.org/lung_tracker/) (accessed on 28 April 2026).

## 4. Smoking and Other Exposures Contributing to COPD

Exposures have long been recognized as a cause of chronic obstructive pulmonary disease. Considerable research over the last century has contributed to and resulted in disease prevention and advanced understanding of pathogenesis. Cigarette smoke has been the most important exposure studied, although other exposures are well-recognized. These studies and a rich ongoing field of investigation have highlighted the complexity and heterogeneity of both relevant exposures and of COPD. While other exposures are recognized as contributing to COPD, smoking has dominated to such a degree that some have stated, incorrectly, that smoking is THE cause of COPD. An unfortunate result of this thinking is that most clinical trials of COPD and many observational studies have been limited to smokers and ex-smokers. While a focus on smokers may have been reasonable for some purposes, it has resulted in a relative paucity of information about COPD among non-smokers where other etiologies are present.

### 4.1. Cigarette Smoking

The 1964 Surgeon General’s report Smoking and Health [34] introduced the impact of smoke on non-respiratory disease: “*Bronchitis and emphysema [i.e., COPD], in particular, severely disable large numbers of men of working age and have a considerable effect upon mortality as a direct or contributory cause of death*.” The subsequent chapter focused on the data, accumulated over the prior decades associating increased mortality and morbidity from COPD with cigarette smoking. However, references from Badham dating to 1805 as well as to Laennec’s famous 1819 treatise, referenced in translation, were noted. That these descriptions of what is almost certainly COPD long preceded cigarette smoking led to the statement: “…*it is reasonable to suggest at the outset that cigarette smoking alone is not the only cause of chronic bronchitis and emphysema*.” Nevertheless, the Surgeon General’s report, by correctly highlighting smoking, may have de-emphasized other exposures in studies over the next several decades.

The importance of smoking was further emphasized by the landmark prospective natural history study of Fletcher and colleagues [14]. This study focused on men working in the British postal service at a time when about 70% of British men were smokers. The prevalence of smoking was sufficiently high that the potential role(s) for other exposures was not realistic to assess. Nevertheless, key findings on the rate of lung function loss associated with smoking led directly to the Lung Health Study by Anthonisen and colleagues that demonstrated that smoking cessation slowed lung function loss [35] and that there were benefits of smoking cessation intervention with nicotine replacement therapy [36]. This question was revisited by Kohansal and colleagues using longitudinal data from the Framingham study. The original finding that smoking cessation was associated with a reduction in the loss of lung function was confirmed. Importantly, the benefits of smoking cessation were greatest when cessation was achieved at a younger age. Conversely, cessation among older smokers did not appear to slow lung function loss. This contrasts with the mortality benefits of smoking cessation that are noted even among the elderly [37].

A key implication from the studies of Anthonisen and Kohansal is that the pathogenic processes initiated by cigarette smoking may be reversible but, particularly with prolonged exposure and/or advancing age, may not be reversible. Active investigations into the cellular, inflammatory, immune, and structural mechanisms involved in these processes are ongoing. Importantly, the heterogeneous aspects of COPD are highlighted by the varied responses to cessation. For example, chronic bronchitis appears to resolve in many but not all people [38].

COPD is a heterogeneous collection of conditions that often overlap within an individual patient. Fletcher and colleagues recognized that not all smokers are susceptible [14]. Genetic studies have defined susceptibility factors. Alpha 1 anti-trypsin deficiency, a rare disease, leads to a large increase in susceptibility [39]. Genome-wide association studies have identified many additional genes which increase COPD risk by much smaller degrees [40]. Importantly, these genes are relatively common and appear to have cumulative effects [41]. This suggests that patients with COPD will have many different and overlapping combinations of genetic risks contributing to disease.

Cigarette smoke composition likely contributes to the clinical heterogeneity of COPD. Cigarette smoke contains a myriad of compounds, many of which are recognized toxins and most of which remain untested [42]. In addition, the composition of smoke varies with individual smoking habits and changes during the smoking of an individual cigarette. It is likely that individuals have varying susceptibilities to these many toxins. Additionally, the composition of cigarette smoke has changed over decades as cigarettes have been modified by manufacturers [43]. This variability may have contributed to changes in the clinical manifestations of COPD over time which, of course, are also influenced by changes in treatments and other comorbidities. This is likely to lead to different toxin histories among smokers of different ages and to complicate a comparison of clinical studies conducted during different decades.

### 4.2. Other Exposures

While cigarette smoking has attracted the most attention, it has been recognized that other occupational [44] and environmental [45,46] exposures also contribute to COPD. Studies demonstrate that these exposures may have mechanisms distinct from smoking. For example, a report from the MESA Lung Study demonstrates the impact of air pollution on progression of both emphysema and loss of FEV1 that was present even when adjusted for smoking and passive smoke exposure [47]. This additive effect is not likely to be simply a ‘dose’ effect as the amount of total exposure to cigarette smoke dwarfs that in ambient air pollution. It is much more likely that the differing compositions of smoke and air pollution are leading to different pathogenetic processes. Of course, these processes may have additive or synergistic consequences.

Exposure to indoor air pollution resulting primarily from biomass heat sources has been demonstrated to lead to COPD [48]. Airway disease is a much more prominent feature of COPD from this etiology than from cigarette smoke [49]. Electronic nicotine delivery systems (ENDSs) are associated with airways disease and bronchitis [50]. ENDSs have been widely used for a relatively short time. While initial studies suggested a five-year duration was insufficient to lead to COPD [51], more recent studies suggest that ENDSs do lead to COPD progression [52].

### 4.3. Moving Forward

Recognition that cigarette smoking is a major cause of COPD has been a major advance with well-documented health benefits. Social efforts to control smoking have reduced smoking burden both by reducing initiation and by increasing cessation [53]. Medical approaches to foster cessation are effective and widely available, although probably under-used [54]. Many questions remain, however, about the pathophysiology of smoking-induced lung disease and the varied responses that contribute to clinical heterogeneity. Control or air pollution has likely had a positive impact as well. Novel exposures, e.g., to ENDSs, are newly emerging hazards. COPD must be recognized as a disease that is much more than a disease of smoking.

## 5. Thinking Outside the Box: The Systemic Nature of COPD

For years, COPD was understood as “a self-inflicted disease by tobacco smoking that occur in older males and characterized by an accelerated decline of lung function with age” and that it predominantly affected the lungs [14]. Research over the past 20 years has demonstrated the systemic nature of COPD including cardiovascular, metabolic, psychiatric, and neoplastic diseases [2]. Thus, the “box” of COPD being just a respiratory disease is no longer true. We now know that COPD shares syndemic origins with other multimorbid diseases, sharing genetic and environmental risk factors [55], and that the appropriate management of COPD requires the search and eventual treatment of these extrapulmonary diseases in a personalized manner [56].

The importance of multimorbidity is recognized in treatment strategies, such as those published by the Global Initiative on Chronic Obstructive Lung Disease (GOLD), which note that comorbid diseases “may have a significant impact on disease course” [2]. Specific guidance on how COPD and its associated comorbid diseases are best addressed, however, are lacking, with statements like “In general, the presence of comorbidities should not alter COPD treatment and comorbidities should be treated per usual standards regardless of the presence of COPD” [2]. This belief, however, may change with additional research and newer therapies. For example, both moderate and severe exacerbations of COPD increase the risk of cardiovascular events post-exacerbation [57]. The specific reasons for this are unclear, but it is possible that the use of short-acting bronchodilators and/or systemic steroids, current “standard” treatments in the treatment of exacerbations, may be adding to this risk. Other studies have demonstrated a doubled risk of mortality among people with obstructive airways diseases [58] who overuse short-acting bronchodilators. Thus, therapies that decrease exposures to short-acting bronchodilators might decrease mortality.

The use of biologic therapies reduces exposures to short-acting beta agonists and systemic steroids, with one study demonstrating a 28% reduction in the former and a 55% reduction in the latter [59]. A recent study in an asthma population demonstrated that patients treated with anti-interleukin (IL)-5/IL5 receptor (IL5R) therapies (mepolizumab and benralizumab) had a significantly lower risk of mortality (adjusted hazard ratio [aHR] 0.35, 95% confidence interval [CI] 0.29–0.42), in addition to reductions in cardiovascular events compared with non-biologic users [60]. The future treatment of COPD exacerbations may need to incorporate cardioprotective strategies to decrease the risk of cardiovascular events in the peri-exacerbation period.

Another example of how a comorbid disease affects COPD can be seen in lung cancer. The United States Prevention Services Task Force recommends “*annual screening for lung cancer with low dose computed tomography scan in adults aged 50 to 80 years who have a 20 pack-year smoking history and currently smoke or have quit within the past 15 years*” [61]. One study of over 16,000 current or former smokers found that nearly 20% had “undiagnosed COPD” (presence of symptoms, no previous COPD diagnosis, and airflow obstruction) [62]. This demonstrates how screening for a comorbid disease like lung cancer can detect people with COPD, leading to earlier intervention.

Thinking “outside the box” for how COPD has been previously viewed and treated should lead to better outcomes in our patients. We now understand that the term “COPD” is too simplistic, as it extends well beyond the lungs to include multiple organ systems. This new understanding opens novel opportunities for prevention and early intervention [63].

## 6. What a Great Idea! Let’s Ask the Patients How They Feel: Patient-Reported Outcomes (PROs) for COPD

### 6.1. Asking the Patient

Early attempts to understand the COPD patient’s perspective of how they felt typically consisted of subjective observations during the physician assessment pertaining to their level of dyspnea. Arguably, the first objective dyspnea measure was reported by C.M. Fletcher (1952) in the Proceedings of the Royal Society of Medicine, based on the 1940 experience of Welsh coal miners [64]. This measure, the Medical Research Council (MRC), is used to objectively rate subjective symptoms and consists of five questions (rated 1–5) which evaluate the degree to which dyspnea impairs daily activities. Still in use today but modified to a 0–4 scale, the Modified MRC (mMRC) is still considered by many to be the gold standard for measuring dyspnea.

### 6.2. Interest Grows in How COPD Affects Patients

As research in COPD increased, more treatment options were available to reduce the burden of this disease. Questionnaires were developed beyond the evaluation of the symptom of dyspnea to guide treatment. However, their main use was in the evaluation of treatment effects (pharmacologic, rehabilitation, surgical, etc.), on groups of patients, rather than routine clinical management of individuals. Examples include the Chronic Respiratory Disease Questionnaire (CRQ) [65] and the Saint George’s Respiratory Questionnaire (SGRQ) [66]. These measures focus on domains that would impact the patient’s overall health-related quality of life, e.g., symptoms of dyspnea and fatigue, the impact of dyspnea on daily activities, and the emotional/psychological effect of COPD. These outcome measures changed the focus from physiologic parameters (such as the forced expiratory volume in one second (FEV1)) to areas of importance to the patient.

### 6.3. Dyspnea Is Complicated

In the 1990s, patients with a variety of conditions affecting breathing were asked to describe how they felt when they were breathless [67]. Interestingly, the descriptors COPD patients chose (gasping, hunger for air, and breathing requires effort) were often different than individuals with asthma or vascular conditions. It became evident that COPD patients experienced unique sensations from others with dyspnea. Additionally, it was found that COPD patients with the same degree of airway obstruction varied in the degree of impairment they experienced with day-to-day activities or the severity of dyspnea. The 1999 [68] and (updated) 2012 [69] statements on dyspnea by the American Thoracic Society outlined how dyspnea can be measured in three different domains: (1) sensory-perceptual; (2) affective distress to the patient; and (3) impact or burden of dyspnea.

Most dyspnea questionnaires only target one of the above three potential areas that may affect a patient. Confounding this lack of sensitivity in measuring dyspnea from one patient to another, Yorke et al. [70] reported how the MRC instrument, the “gold standard” for measuring dyspnea, could be confusing to patients. For example, Grade 0 asked about restrictions in “strenuous” exercise. Some patients indicated they did not do “strenuous” exercise or were unclear what specific exercise was being referred to by “strenuous”. Also, some did not understand if Grade 4 (“Too breathless to leave the house or breathless when dressing or undressing”) was a single experience or two experiences [70] (pg. 2293). Patients viewed these as distinctly separate activities.

### 6.4. So, Let’s Go Back and Ask the Patient

Given that all COPD patients are not alike, how does one target their unique concerns? Artificial Intelligence (AI) has the potential to gather this unique information without the time burden of a questionnaire, but with likely more precision. Early uses of Computerized Adaptive Testing (CAT) with Item Response Testing (IRT) were used with the Patient-Reported Outcomes Measurement Information System (PROMIS^®^) dyspnea measure. The promise of CAT with built-in IRT has increased rigor and confidence with shorter, more adaptive questionnaires. This work, which is currently underway, promises a simple approach which could be incorporated with high rigor and confidence in ongoing clinical care [71,72]. AI, whose strength is pattern recognition, may be an ideal tool to increase the accuracy of PRO measures. Initial evidence of using AI to estimate or predict PROs has been carried out with COPDGene and Eclipse initiatives, which linked CT features with PROs such as the SGRQ, without the burden of collecting data from questionnaires [73].

Despite efforts to advance the field, we may still be missing the important potential of machine learning (ML), PRO remote monitoring, and predictive algorithms that can provide alerts and management suggestions based on the patients’ own perceptions. While AI refers to machines imitating human intelligence, ML is a subset of AI that “teaches” computers to find patterns and make predictions [74]. By accumulating input from the patient over time, ML can “learn” about the patient’s health status, assisting patients who often have difficulty expressing fluctuations in their current state to their provider. In this way, remote PRO monitoring and ML could potentially help with recognition of exacerbations [75,76]. Conceivably, a patient could be monitored on a regular basis via watch, ring, or cellphone, as currently done with fall monitoring. Patients can validate (or mark as erroneous) an event related to their daily activities, breathing status, cough, or wheeze that is bothersome. This information can go the next step in helping the provider and patient develop an *action plan*, specific to the patient’s needs, rooted in their reported outcomes.

Some initial work in this area has used CAir desk (remote monitoring devices), where hybrid virtual coaching has occurred with improvements in physical activity and decreases in symptom burden [77]. We are just at the beginning of what could be done with remote monitoring, AI, and ML to improve care and health-related quality of life in COPD patients in the coming years.

While researchers and providers should continue focusing on PROs, utilizing AI may allow patients to tell their stories without subjecting them to burdensome questionnaires. More specifically, if the goal is accumulating more accurate PRO data with less burden, adopting a COPD-specific CAT/IRT instrument is in order. If the goal is improving outcomes, pairing AI risk-prediction or chatbot education with predefined, PRO-targeted actions (e.g., referral to pulmonary rehabilitation and inhaler technique coaching) at fixed intervals is in order. The future is upon us, as we need to be proactive in determining how to use AI and ML to optimize individualized patient care.

## 7. Exacerbations

Although the “*O*” of COPD, indicating the presence of airflow obstruction is integral to its definition, focusing solely on this one aspect is far too simplistic. The course of this disease is frequently punctuated by exacerbations which negatively impact the progression of airflow obstruction [78], symptoms [79], health-related quality of life [80], health care utilization [81], and mortality risk [82]. Before the full impact of exacerbations was understood, earlier definitions focused simply on its cardinal symptoms, including dyspnea, cough, and sputum purulence beyond day-to-day variability [83]. Respiratory symptoms with exacerbations can have a relatively sudden onset (over a one-day period, 56%) or a more gradual onset over days (44%) [84].

More recent definitions of COPD reflect changes in the concept as more knowledge of the disease has accrued [85]. The Global Initiative for Chronic Obstructive Lung Disease (GOLD) committee recognized the impact of exacerbations on the progression of the disease, adding this sentence to its definition: “*Exacerbations and comorbidities significantly contribute to the overall severity in individual patients.*” [2].

An attempt at an updated definition of exacerbations came from a committee of 17 expert clinicians in the field who recognized the importance of the “acute burst of airway inflammation” [85] due to etiologies such as respiratory infections and environmental pollutants. This definition, referenced as The Rome Proposal, is as follows: “*In a patient with COPD*, *an exacerbation is an event characterized by dyspnea and/or cough and sputum that worsen over ≤14 days*, *which may be accompanied by tachypnea and/or tachycardia and is often associated with increased local and systemic inflammation caused by airway infection*, *pollution*, *or other insult to the airways.*” [85]. This definition, therefore, brings in not just increased local and systemic inflammation but also etiologic agents, physiologic changes, and the time course of the event. Chronic lung inflammation in COPD is often characterized by type 1 inflammation, characterized by neutrophilic inflammation that is mediated by such proinflammatory mediators as tumor necrosis factor and interleukin-6. However, in up to 40%, type 2 (Th-2)-mediated airway inflammation is present, as evidenced by increased eosinophils in the airways or blood. This type of inflammation is associated with a greater risk of exacerbations, and direct pharmacologic therapy for this endotype is available, such as inhaled corticosteroids [86].

Another important concept has been the recognition of the marked variability in the frequency of exacerbations in COPD, with some never experiencing these events and others with several per year—leading to the recognition of the frequent exacerbator phenotype [87]. The strongest factor predicting a COPD exacerbation is the history of multiple exacerbations, such as two or more exacerbations in a year that lead to increased health care utilization (HCU). The HCU requirement, which can include ramped-up pharmacologic therapy, hospitalization, or urgent/emergency treatment, is necessary, since with in-depth reporting of symptoms [88], many exacerbations go unrecognized by patients. Adding to the negative impact of exacerbations is temporal clustering: one or more exacerbations are more likely to be followed by subsequent ones, typically leading to prolonged inflammation [89].

Since exacerbations have such a profound, negative impact, emphasis on mitigating their effects on the COPD patient (especially for the frequent exacerbator) is good medical practice. This would include preventative strategies, such as vaccinations; smoking cessation interventions; increased physical exercise; dual-bronchodilator therapy; and—in those with Th-2 inflammation—inhaled corticosteroids in combination with bronchodilators as well as biologic therapies. Likely of benefit are collaborative self-management strategies [90] (patient and health care professional) which are considered part of integrated care [91]. These strategies focus on the early recognition of the exacerbation, enhanced communication with the health care professional should an exacerbation occur, prompt initiation of exacerbation therapies through a self-management plan (such as oral corticosteroids and antibiotics), and ongoing follow-up with the health care provider. A recent, updated review of self-management interventions for COPD shows that it has a major, positive effect on health-related quality of life and a small effect on health care utilization but no differences observed in respiratory-related mortality and all-cause mortality [92]. Finally, pulmonary rehabilitation initiated shortly after a COPD exacerbation significantly improves health-related quality of life and exercise capacity. It also reduces hospital readmissions and mortality [93].

## 8. Hyperinflation in COPD

Expiratory flow limitation (EFL) has long been established as the pathophysiological hallmark of COPD, and its underlying mechanisms are well understood [94,95,96]. Measurement of the ratio of forced expiratory volume in 1 s (FEV_1_) to forced vital capacity (FVC) provides a crude surrogate for diagnosis [2], while FEV_1_% predicted or, more recently, z-scores [97], indicate overall “disease severity” [98]. However, exclusive reliance on EFL measures for diagnosis, assessment, and management planning has limitations [99].

We now know that there is substantial heterogeneity of physiological impairment among patients with similar FEV_1_ values [100]. Moreover, FEV_1_ may not consistently serve as a robust physiological biomarker, as it does not reliably correlate with key clinical outcomes, including symptom burden, health-related quality of life, and exercise tolerance [100]. Sadly, poor bronchodilator reversibility, as judged by a minor or absent change in FEV_1_ [101], has led to the notion of “irreversible” airway obstruction and has historically engendered a degree of therapeutic nihilism. This state of affairs has prompted clinicians to consider other functional markers that could complement FEV_1_ in assessing the true nature and extent of disease severity [102].

### 8.1. The Physiology of Hyperinflation and Its Clinical Implications

Physical signs of lung hyperinflation and structural emphysema on imaging have long been recognized as major pathophysiological features in large subsets of patients [4]. Indeed, it has been long recognized that this clinical phenotype is more likely to be associated with severe breathlessness [103]. Seminal mechanical studies that measured pressure/volume relations of the relaxed respiratory system demonstrated that reduced lung elastic recoil pressure of the emphysematous lung was the main cause of what is termed “static” lung hyperinflation [95,104]. Unfortunately, for many years, the close association between hyperinflation and destructive emphysema meant it was not considered a realistic therapeutic target [105]. Thankfully, this notion is no longer tenable.

Physiologists who first elucidated the mechanisms and adverse consequences of EFL also highlighted the phenomenon of “dynamic” hyperinflation, which refers to the transient, variable increase in end-expiratory lung volume (EELV) above its baseline resting value [106,107]. EELV is used interchangeably with functional residual capacity (FRC) in this document. It became clear that hyperinflation, measured at rest (using plethysmography or gas-dilution techniques), encompassed both static and dynamic components. In other words, resting lung hyperinflation may be due to reduced lung elastance; expiratory flow limitation effects; or, as is often the case, both [108,109]. In the presence of EFL, acute increases in breathing frequency (with reduced expiratory time) further delay lung emptying, thereby acutely elevating EELV above its variably elevated baseline. Static plus dynamic hyperinflation causes excessive mechanical (elastic) loading of the inspiratory muscles and increased intrinsic positive end-expiratory pressure (PEEPi) [110,111]. Thus, it functionally weakens the diaphragm and accessory inspiratory muscles [112] and erodes the available inspiratory reserve volume (IRV), thereby significantly compromising cardiovascular function due to increased positive intrathoracic pressures [113,114,115].

**Figure 5:**
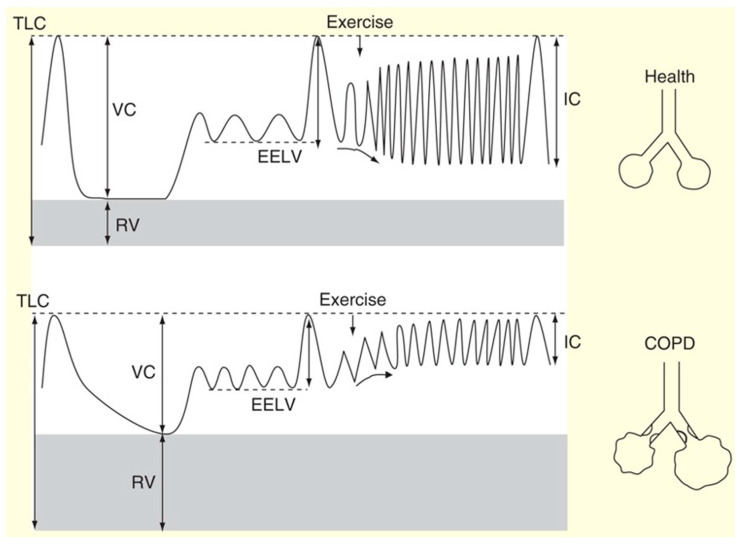
The effects of exercise in healthy individuals and patients with COPD.

Over the last few decades, physiological studies informed clinicians that episodes of dynamic hyperinflation regularly occurred in the daily lives of their patients, when, for example, EFL worsened (exacerbation and bronchospasm) [116,117] or when bouts of tachypnea suddenly occurred (e.g., with physical activity, anxiety/panic, acute hypoxemia, etc.). Hyperinflation forces patients to breathe on the higher, less compliant portion of the sigmoidal pressure/volume curve of the respiratory system, where greater inspiratory muscle effort and inspiratory neural drive are needed to maintain ventilation [96,118]. With exercise, as EELV increases, patients breathe near total lung capacity (TLC), resulting in a growing disparity (or dissociation) between heightened inspiratory neural drive and the progressive inability to appropriately expand tidal volume (VT). This phenomenon of neuromechanical dissociation (NMD) of the respiratory system [99] is closely linked to breathlessness [108] and its main qualitative dimensions (“unsatisfied inspiration” or “can’t get enough air in”) (reviewed in [119]) (Figure 5).

Dynamic hyperinflation during exercise, signaling small airway dysfunction, EFL, and pulmonary gas trapping [120], has been observed in smokers with the chronic bronchitis phenotype [121], in those with mild COPD [122], and even in symptomatic smokers who do not meet COPD diagnosis criteria [123]. Collectively, these studies confirm that previously overlooked dynamic mechanical factors during exercise consistently contribute to dyspnea, ventilatory restraints, and exercise intolerance in many patients across the COPD spectrum (recently reviewed in [124]).

### 8.2. Dynamic Hyperinflation and Its Clinical Consequences

The link between hyperinflation and breathlessness was facilitated by experimental confirmation that resting inspiratory capacity (IC) was a reliable surrogate for FRC [102,109,125]. Low resting IC signifies hyperinflation, provided that there is no significant inspiratory muscle weakness from another cause [126]. Importantly, resting IC was shown to be independently associated with relevant clinical outcomes, including increased dyspnea burden, reduced exercise capacity, poor health-related quality of life, increased propensity for exacerbations/ hospitalizations, more rapid disease progression, and increased mortality [127,128,129,130]. These associations held after accounting for FEV_1_ and other relevant variables [128,131]. This new knowledge supported a solid rationale for therapeutic targeting of hyperinflation to improve these clinical outcomes.

### 8.3. Mitigating the Negative Effects of Hyperinflation

The question naturally arose whether it was possible to therapeutically reduce hyperinflation to a degree that would improve exertional breathlessness, thereby facilitating increased physical activity [132]. The answer was a resounding YES! Studies confirmed that a resetting of resting EELV closer to its predictive value could be achieved by bronchodilator therapy, which decreased airways resistance, promoting enhanced lung emptying [133,134]; by breathing pattern manipulation that increased time spent in exhalation on mechanical ventilation, including pursed lip breathing [135]; or in selected individuals with advanced emphysema, by surgical or endoscopic lung volume reduction, that increased lung elastic recoil pressure of the remaining lung, promoting better lung emptying [136,137,138,139]. A concise overview of the effects of selected pharmacological and non-pharmacological interventions on hyperinflation is shown in Table 1.

### 8.4. Therapies to Reduce Hyperinflation

Long-acting bronchodilators can provide sustained, additive effects on lung deflation, compared with each component alone [140,141]. Twenty-four-hour “pharmacological” lung deflation is a desirable goal, as nighttime hyperinflation, with increased morning EELV on awakening (and accompanying aggravated symptoms) [142], is prevalent in COPD populations and should not be ignored [142]. It is now also recognized that relatively small bronchodilator-induced increases in resting IC (~0.2–3 L) are associated with lower symptom burden and greater exercise endurance [143].

Following optimal bronchodilator therapy, encouraging physical activity is arguably the most powerful intervention for improving dyspnea and quality of life in symptomatic patients. Engagement in supervised exercise training, preferably within rehabilitation programs, is strongly advocated. While improvements following exercise training are multi-factorial [144], it has emerged that increased breathing efficiency, with reduced respiratory rate (and ventilation) and concomitant reduction in dynamic hyperinflation, is an important mechanism of benefit [145,146,147,148].

Oxygen supplementation, by reducing ventilatory requirements and slowing respiratory rate (in a dose-dependent manner), increased submaximal IC and improved dyspnea and exercise endurance times, in some but not all patients [149] (also reviewed in [150]). Interestingly, bronchodilator therapy combined with oxygen supplementation showed additive effects on exertional dyspnea, attributable to the combined effects of higher IC and lower inspiratory neural drive [151]. Using the same unitary physiological rationale, investigators reported that other experimental adjuncts to exercise training, such as mechanical assistance (low-level continuous positive airway pressure (CPAP), pressure support, and their combination (i.e., bi-level positive airway pressure (BiPAP)), successfully increased IC, and improved breathlessness and exercise endurance times in a subset of patients with severe COPD [152,153,154,155]. However, the overall utility and efficacy of these adjunct measures have not yet been established [156].

The importance of severe chronic hyperinflation as a key therapeutic target was bolstered by studies demonstrating that carefully selected patients with severe air trapping (RV > 150% predicted), upper lobe emphysema, and poor exercise tolerance can benefit from lung volume reduction surgery [157], resulting in improved lung elastic recoil pressure and expiratory flow rates, inspiratory muscle function, and cardiovascular function [158,159,160]. Important clinical benefits, including improved chronic breathlessness, exercise tolerance, and quality of life and reduced long-term mortality, were confirmed in carefully selected patients [158]. Subsequently, similar physiological and clinical benefits, except for reduced mortality, were reported in highly selected patients with intact interlobar fissures or the absence of collateral ventilation with incapacitating breathlessness after endoscopic volume-reduction procedures (endobronchial valve placement) [136,138,139].

### 8.5. Exacerbations and Hyperinflation

Worsening EFL and “acute-on-chronic” hyperinflation, which can critically compromise respiratory muscle and cardiovascular function, have now become the defining features of severe COPD exacerbation [161]. Convincing evidence that the effects of hyperinflation could be successfully mitigated emerged from studies in intensive care settings of patients with severe hyperinflation and respiratory failure [162,163]. Individualized titration of low-level CPAP to counterbalance PEEPi, added pressure support, and breathing pattern manipulations (to promote lung emptying) partially ameliorated the adverse mechanical and sensory effects of severe hyperinflation [164]. Later, non-invasive ventilation strategies using mask-based BiPAP were shown to be effective in preventing intubation, shortening hospitalization duration, and reducing mortality rates in patients admitted with severe exacerbations [165].

## 9. Pharmacologic Therapy for COPD

An important event in the history of COPD was a 1959 CIBA guest symposium where the characterization of the disease included the combination of chronic bronchitis, airflow obstruction, and emphysema [21]. This symposium emphasized the role of airflow obstruction in the pathophysiology of COPD, and this led to the identification of the role of hyperinflation in the development of dyspnea. Based on these findings, early pharmacological therapy in COPD revolved around the use of short-acting bronchodilators, such as salbutamol [166], used initially orally and then as inhaled formulations, with the development of improved inhaler devices.

The seminal work of Fletcher and Peto in 1977 emphasized that COPD is a progressive disease associated with lung function decline over time, but they also showed that FEV1 decline is variable among COPD subjects, with a number of trajectories [14]. They also indicated that infections or exacerbations did not contribute to this lung function decline. Thus, the target of pharmacological therapy in COPD switched to the effect of corticosteroids on lung function decline. Inhaled corticosteroids had been shown to be effective in asthma and were often prescribed in COPD based on mainly short-term studies. So, in the late 1990s, several studies were organized to evaluate the role of inhaled corticosteroids in disease progression. The most well-known trials were EUROSCOP [167] with inhaled budesonide and ISOLDE [168] with inhaled fluticasone. ISOLDE recruited smokers with more severe COPD, while EUROSCOP recruited smokers with milder disease. Both trials showed no overall effect on FEV1 decline over a 3-year follow-up period, but the ISOLDE study showed a reduction in COPD exacerbations that changed the focus of pharmacological development in COPD.

At the same time as the ISOLDE study, there was increasing interest in the nature of COPD exacerbations [78]. Contrary to the work of Fletcher and Peto, several groups showed that exacerbations not only affect health status, hospitalization, and survival but also importantly disease progression [80]. Some COPD patients are prone to frequent exacerbations [169], and future COPD trials became focused on these higher-risk COPD subjects.

Inhaled corticosteroids (ICSs) were combined with long-acting beta agonists (LABAs) (ICSs/LABAs) in a single inhaler, and studies showed a reduction in COPD exacerbations that was greater with the combination than with the individual inhaled components. Any therapy that reduces exacerbations should reduce mortality, but the TORCH study designed to detect a mortality benefit with ICS/LABA just missed the primary end point [170]. A new class of bronchodilators was introduced with specific inhaled long-acting anti-muscarinic agents (LAMAs) such as tiotropium that not only were effective in reducing hyperinflation and dyspnea but also reduced exacerbation frequency [171]. Dual bronchodilators with LABAs/LAMAs combined were developed and showed improved symptomatic benefit and advantages with respect to exacerbations compared to ICS/LABA combinations. Thus, the GOLD strategic document for the management of COPD suggested that dual bronchodilators are initial therapy in COPD, while ICSs/LABAs are now not indicated in COPD, unless there is concomitant asthma [2].

The next step was to combine all three agents in one inhaler as triple inhaled therapy, and studies showed that with triple therapy, prevention of exacerbations was more effective than with ICS/LABA or LABA/LAMA combinations [172,173]. Other benefits included improvement in lung function, symptoms, and health status. As these studies lasted only one year, COPD mortality rates were less than in the TORCH study, but triple therapy and ICS-containing therapies were associated with a mortality benefit. Other therapies for exacerbation reduction have included long-term macrolide therapy [174,175] and also phosphodiesterase 4 (PDE4) inhibitors, such as roflumilast [176] (17) and the novel inhaled phosphodiesterase 3 (PDE3) (PDE3/PDE4) inhibitor, ensifentrine [177].

A more recent major development in the pharmacologic management of COPD was the reanalysis of the earlier ICS trials with respect to the level of blood eosinophils (a marker of type 2 inflammation) [178]. It was shown that ICS therapies were more effective when blood eosinophils were elevated (above 100/uL), and the effect increased with increasing levels of type 2 inflammation. Early studies of biological agents in COPD, especially with anti-interleukin-5 (anti-IL5) agents, showed no overall effects on exacerbation reduction, but once the data was analyzed by blood eosinophil levels and new trials designed, exacerbation reduction was seen in those individuals with higher blood eosinophil levels [179,180]. Additionally, trials of dupilimab (anti-IL4/IL13) were also performed in COPD subjects with type 2 inflammation and blood eosinophils above 300 uL, with a respectable reduction in exacerbations [181]. Other properties of biologic agents are being assessed such as their effect on mucus plugs, and this will likely lead to the development of anti-mucus agents. However, type 1 inflammation, which is more common in COPD, has been more difficult to target due to the complexity of pathways.

Finally, there have been important advances in the understanding of the mechanisms of disease progression in COPD and the nature of exacerbations. With these advances, COPD pharmacological therapy has evolved from symptomatic therapy with bronchodilators to identification of higher-risk individuals and targeting type 2 inflammation. However, only up to 25% of COPD subjects have type 2 inflammation. The challenge for future drug development is to identify biologic agents and novel compounds that target most COPD subjects with type 1 inflammation.

## 10. Long-Term Oxygen Therapy…Well Established but in Turmoil

### 10.1. Historical Perspective

One might suppose that a therapy with which we have almost a century of experience would have the kinks worked out. One would be wrong! For skimming lightly over the history of clinical use of supplemental oxygen, one couldn’t do better than to read a delightful recent biography of Alvan Barach [182], a physician who practiced in New York roughly from the 1920s to the 1970s. From oxygen tents and oxygen rooms for hospitalized patients in the 1920s to early use of portable oxygen supplies in the 1950s, Dr. Barach’s work shows what an ingenious individual can do to develop a valuable therapy.

The next historical milestone that shaped oxygen therapy has to be the performance of two randomized clinical trials in the late 1970s, one in the US (the Nocturnal Oxygen Therapy Trial, NOTT [183]) and one in Britain (the Medical Research Council, MRC, Trial [184]). Though not designed primarily as mortality studies, these two studies, led by Drs. Thomas Petty and David Flenley, showed that COPD subjects with resting severe hypoxemia gained dramatic survival advantage from supplemental oxygen: the more hours of oxygen per day, the better the survival. These trials set the stage for the present practice of oxygen therapy in which, although up-to-date figures are hard to obtain, over a million patients in the United States receive long-term oxygen therapy (LTOT) at a cost likely well in excess of $1 billion per year. Beyond the U.S., it is estimated that 9 million people annually require long-term oxygen therapy (LTOT) for chronic respiratory diseases. Bridging this gap would require an estimated $34 billion over the next five years, but it is considered a highly cost-effective investment.

### 10.2. Toward Better Oxygen Devices

Drawing oxygen out of the ambient air is manifestly the most desirable way to provide long-term oxygen to those who need it. Providing compressed oxygen cylinders or liquid oxygen as a routine modality is inefficient and costly as both require delivery of oxygen to the home. Liquid oxygen, in particular, requires infrastructure that makes it impractical to supply a modest number of patients distributed over wide geographic areas. The problem is that existing oxygen concentrator technology is often incapable of providing sufficient oxygen for the minority of oxygen-requiring patients who require high oxygen flow rates. This is especially a concern for the provision of ambulatory oxygen, where an undesirable tradeoff between rate of oxygen supply and weight of the device exists. The technology used in oxygen concentrators is based on a principle reduced to practice roughly 50 years ago. It requires cyclical adsorption of nitrogen (80% of air volume) by zeolite, leaving near pure oxygen behind [185]—arguably an inefficient process. New, more efficient, technologies for oxygen concentration could change the landscape of LTOT provision, most importantly allowing provision of higher oxygen flow from lighter devices, more conducive to use during ambulation by frail LTOT patients.

Further complicating the situation for patients trying to obtain portable oxygen supplementation devices at a reasonable price, portable oxygen concentrators are available from on-line sellers (without a prescription). These devices are considerably cheaper than prescription devices; however, a recent study of three devices obtained on-line demonstrated that two of the three did not provide clinically useful generation of oxygen [186]. Buyers beware!

### 10.3. Who Really Needs Long-Term Oxygen Therapy?

Hypoxemic patients who receive supplemental oxygen tend to increase exercise endurance and reduce exertional breathlessness [187]. But, oxygen supplementation is an expensive and intrusive therapy, and we would hesitate to provide it widely if these were the only benefits. In a perverse way, the 45-year-old NOTT and MRC oxygen trials [183,184] are a straitjacket: we must provide LTOT to severely hypoxemic patients because life prolongation was demonstrated in these two trials. Much has changed in treatment paradigms in the past 45 years, but ethical considerations continue to prohibit a randomized trial of such patients in which a subgroup is denied LTOT. Nevertheless, well-designed trials have been performed relevant to the question of LTOT need, e.g., in COPD patients with moderate resting hypoxemia [188,189], in patients with exercise-only hypoxemia [188], and while providing patients with severe resting hypoxemia supplemental oxygen fewer hours per day [190]. None of these trials have been able to detect long-term important benefits, including lower hospitalization rates or better survival.

A particular focus might be on those with exertional hypoxemia (with or without resting hypoxemia). Utilizing ambulatory oxygen is especially onerous for the frail and elderly. Most of these patients are quite sedentary and might ambulate for only an hour or two a day. As practitioners, can we be dogmatic in insisting that they utilize their oxygen whenever they ambulate; can we be confident that their prognosis will be impaired if they do not?

### 10.4. Reconsidering Oxygen Prescribing Criteria

The core threshold for considering oxygen supplementation has been a resting arterial oxygen partial pressure of 55 mmHg. This can be directly traced back to the protocol design criteria of the NOTT and MRC trials, which were selected somewhat arbitrarily. This often has been transposed to a pulse oximeter reading of 88%, which (in addition to the inaccuracies inherent in pulse oximetry) is an inexact representation of a partial pressure of 55 mmHg [191]. Worse, LTOT is often prescribed based on measurements made in hospital during a COPD exacerbation and not always recertified after full recovery. Evidence has been presented that, on re-evaluation, many patients receiving LTOT do not meet prescribing criteria [192,193].

But, a more basic issue might be raised. Arterial oxygen partial pressure has only a remote relation to oxygen levels in the body’s metabolizing tissues, the site at which hypoxia does damage to the organism. An index of tissue hypoxia would be the more logical basis for prescribing supplemental oxygen. A recent publication [194] points out that biomarkers of tissue hypoxia have been identified; however, none seem to have the potential to be useful clinically. Nonetheless, certain biomarker patterns have been speculated to suggest oxygen need (or a lack of oxygen need) independent of arterial oxygenation. Further biomarker research and subsequent clinical trials seem likely to be productive future endeavors.

In summary, oxygen supplementation is of unquestioned value to carefully selected patients with a range of lung diseases. Surprisingly, though, despite its long history of use, more work needs to be done to achieve maximum benefit.

## 11. Non-Pharmacologic Management of COPD: The Magic of Rehabilitation

Magic occurs when actions result in dramatic, exciting, and sometimes unexpected changes. When it comes to pulmonary rehabilitation (PR), those who have done it will tell you about its transformative impact on their daily lives.

### 11.1. Historical Foundation of Exercise

**Figure 6:**
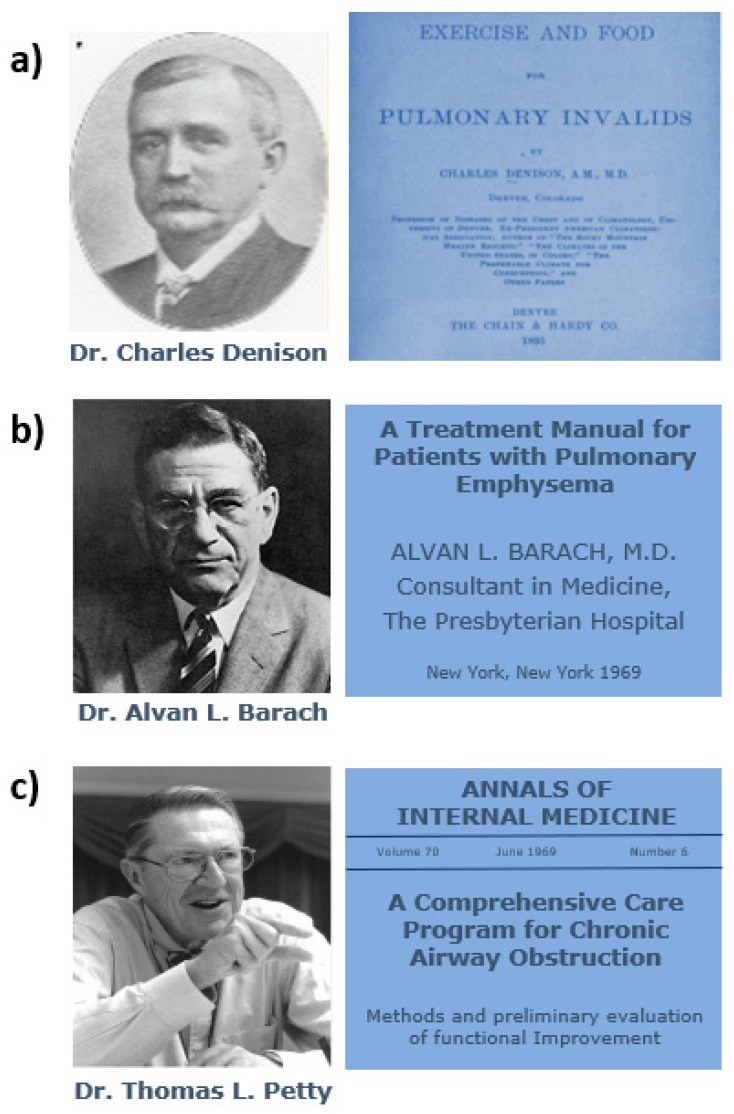
Three pioneers in pulmonary rehabilitation. (**a**) Charles Dennison, who advocated in 1895 that exercise could improve health in patients with respiratory diseases, provided the lungs were not acutely inflamed. (**b**) Alvin Barach, who contributed to the early development of supervised exercise and supportive care approaches for patients with chronic obstructive pulmonary disease (COPD). (**c**) Tom Petty, who helped establish the foundations of modern pulmonary rehabilitation through the integration of exercise training, education, and psychosocial support for patients with COPD.

The idea that exercise could improve health in those with respiratory conditions was advocated in 1895 by Charles Dennison, a Denver Professor of Diseases of the Chest and Climatology [195] (Figure 6a), himself a pulmonary invalid from phthisis, who noted that exercise improved health in a way that would complement existing remedies, provided the lungs were not acutely inflamed. Some 75 years later, Alvin Barach [196] (Figure 6b) and Tom Petty [197] (Figure 6c) published the forerunners of modern PR, identifying supervised exercise, education, and psychological and social support as key to improving function in those whose lives were affected by COPD.

### 11.2. Progress and Recognition

It is remarkable how far PR has come over the last 30 years. It is now recognized by professional respiratory societies around the world as the standard of care for chronic respiratory disease. This evidence-based achievement has resulted from the work of many dedicated colleagues, including the contributors to this communication.

### 11.3. Early Milestones

A key early milestone was the introduction of simple, valid, reproducible, interpretable disease-specific outcome measures of exercise, such as the 6 min walk [198] and the shuttle walk [199] tests. Together with measures of health-related quality of life, such as the Chronic Respiratory Questionnaire [65] and the St George’s Questionnaire [66], these measures enabled prospective randomized controlled trials to clarify the improvements in physical function, mood, and health care resource utilization among those with COPD [200,201,202,203].

During this period, it became evident that for patients with COPD, rigorous exercise training could improve peak work rate and maximum oxygen uptake (the gold standard of cardiorespiratory fitness), as well as physiological parameters associated with constant work exercise [204]. A landmark study identified the adaptation of skeletal muscle oxidative and glycolytic enzymes to endurance training, confirming that the peripheral muscle dysfunction in COPD was remediable with supervised exercise [205]. These studies provided a strong physiological footing for PR. A subsequent series of studies from the Netherlands [206] addressed Denison’s other early recommendation for improving the pulmonary patient, clarifying the importance of good nutrition in promoting exercise performance and quality of life, as well as reducing exacerbations in COPD.

### 11.4. Mood, Multidisciplinary Teams, and Expanded Programs

PR is inherently multidisciplinary, relying on collaboration among physicians and allied health care professionals—especially physical therapists, respiratory therapists, and nurses—to optimize patient outcomes. This collaborative approach has enabled PR to be introduced earlier during acute care, such as during critical illness or soon after hospitalization for a respiratory exacerbation [207], with strong evidence supporting its effectiveness in improving function, reducing repeat admissions, and reducing mortality [93]. Program durations have also been extended, with maintenance and repeat programs designed to preserve functional gains and counteract the sedentary lifestyle frequently associated with COPD [208].

Over time, we have developed a much better understand of the impact of mood on physical function in COPD, with good evidence supporting its positive effects on anxiety and depression [209]. As a result, many programs now include mood as part of their outcomes. Another important development has been for patients with severe COPD and co-existing respiratory failure, for whom clinicians can initiate non-invasive ventilation to stabilize the respiratory system during rehabilitation [210,211].

### 11.5. Evolving Content and Delivery Models

Program content has been steadily evolving to include a wide range of exercise, education, and psychological modalities in the rehabilitation toolbox. Exercise options can include regular or interval training, conventional or single-leg cycling, Tai Chi, dance, and land- or water-based physical therapy [212]. Despite the expansion and effectiveness of PR, issues of access and capacity mean that only a small percentage of those eligible receive it. A variety of factors influence program referral, uptake, and completion [213] (Table 2).

In part, these relate to the knowledge of patients and health care professionals about PR, the environment of travel, time, costs, and co-existing conditions; beliefs about the value of PR; and fears that patients cannot do it or will not benefit from it. There are also important social influences of health care professionals and families that may encourage acceptance of PR.

Program diversity has grown to include in-patient, out-patient, community, or virtual PR, with hybrid programs that combine locations for maximum effectiveness. The COVID-19 epidemic prompted many programs to pivot to virtual PR, and they continue to use this modality for reasons of cost and convenience [214,215,216].

### 11.6. Guidelines, Broader Application, and Integrating Care

In recent years, professional respiratory societies around the world have authored guidelines for PR [217,218,219,220] (Table 3), underscoring the importance of this modality to improve health and reduce resource utilization in the chronic respiratory disease population. Whereas initial research focused on COPD, the benefits of PR extend to other respiratory conditions such as interstitial lung disease, suppurative lung disease, and pulmonary hypertension, as well as pre- and post-lung transplantation.

**Figure 7:**
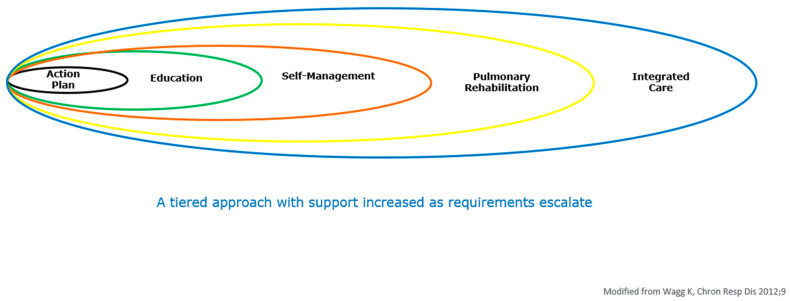
Pulmonary rehabilitation and the spectrum of support. Modified from Wagg et al. [221].

The concept of non-pharmacological management of COPD has shifted from the old notion of standalone, single interventions to that of a tiered model [221] (Figure 7) based on patient need. Those less affected may only require education and an action plan (not PR), whereas others benefit from self-management and a comprehensive exercise intervention (PR). PR plays a central role in the emerging collaborative models of care, which prioritize integrated disease management to prevent clinical deterioration and address co-existing medical and mental health conditions [222,223]. By embedding PR within this broader team-based approach, the respiratory population can fully benefit from the transformative power—the magic—of rehabilitation.

## 12. Evolving Concepts in COPD

Recent years have seen an explosion of excitement and novel research in the field of COPD. As highlighted by several authors in this summary emphasizing the evolution of COPD, significant advances have been made in the following areas: (1) recognition of a broader range of risk factors and exposures; (2) appreciation of the broader spectrum of trajectories by which people develop COPD [2,16,22,224]; (3) the impact of gene and environment interactions over time in COPD pathogenesis; and (4) an increased understanding of the spectrum of comorbidities and systemic manifestations of COPD and their impact on patient outcomes. Novel bench research has fostered an understanding of important pathobiologic mechanisms underlying COPD [26,225,226], including adaptive immune responses to self- or foreign antigens [26], underlying genetic risks and epigenetic changes [26], alterations in lung development and early life events [225], loss of small airways [227,228], pulmonary vascular pruning [229,230], accelerated lung aging [26], autoimmunity [26,231], alterations in the pulmonary microbiome [232], and recognition of the importance of Th2 inflammation among some individuals [232,233,234].

Recent novel work using single-cell RNA sequencing profiles of explanted lung tissue has identified alterations in cellular populations (including aberrant alveolar epithelial type II cells, pulmonary capillary endothelial cells, and macrophages) in advanced COPD compared with non-COPD controls [235], as well as gene alterations and disrupted epithelial barrier pathways in emphysema [236]. Collectively, these advances have led to the recognition of various “phenotypes” and “endotypes” of the disease, and information has emerged regarding biomarkers to identify some of these subgroups [225,226,232,237,238,239]. Accordingly, in recent years, therapies for COPD have become more personalized. Examples of this include lung volume reduction surgery (surgical or endobronchial valve-based) for severe emphysema [157,240,241]; roflumilast (phosphodiesterase-4 inhibitor) to reduce exacerbations among those with severe airflow obstruction and features of chronic bronchitis [176]; inhaled corticosteroids; and most recently, biologic therapies for people with COPD and peripheral blood eosinophilia [232,238]. A distinction has also been made between recommended pharmacotherapies depending on whether patients’ primary issue is dyspnea or experiencing frequent exacerbations. All of this work has set the stage for significant ongoing advances in COPD care for the future.

Several key aspects of further patient characterization are important to note. First, an increased understanding of the pathobiology of COPD acquired via different risk factors/exposures/trajectories and of the extent to which these pathways overlap vs. differ will be essential in order to determine whether differential treatments would be required [22,239,242,243,244,245]. This will need to include gaining knowledge regarding various environmental risks, in particular climate change [246], and the synergistic effects between combinations of risk factors over time [2].

Second, we need an enhanced understanding of epithelial-derived alarmins [e.g., thymic stromal lymphopoietin (TSLP) and IL-33] [238,247], adaptive immunity and Th1-predominant inflammation [248], other non-Th2 inflammatory pathways, and the potential role of FeNO as a useful biomarker for disease categorization [238,249]. Other biomarkers identifying key disease pathways are also needed, especially for individuals with emphysema-predominant disease [226,232,250].

Third, ideally, clinicians and health systems will need to begin monitoring lung function routinely during childhood, to track it sequentially over time, and to identify individuals at risk early in the course of their disease when the opportunity to intervene and mitigate risk is arguably greatest. Moreover, there is a need for better understanding of COPD activity [251].

Fourth, increased knowledge of the factors underlying progression from “pre-COPD” [252,253] and preserved ratio-impaired spirometry (PRISM) [254,255] to having airflow obstruction, as well as the clinical and prognostic relevance of asymptomatic mucus plugs [256,257] and other radiologically identifiable features should help to focus patient follow-up and guide management interventions.

Fifth, given the heterogeneity of COPD and its potential precursors wherein airflow obstruction is not yet present, health care professionals also need to reckon with proposals to redefine COPD. Arguably, the presence of airflow obstruction (the “O” in COPD) may not be integral to the disease definition, and a new diagnostic classification may be in order [22,258]. This may include the presence of respiratory symptoms, impaired respiratory quality of life, impaired spirometry, structural lung abnormalities on CT imaging, frequent exacerbations, rapid lung function decline, and increased mortality risk. The broad implementation of such changes in COPD definition and classification would have major impacts for COPD diagnosis; disease monitoring; and importantly, disease diagnosis coding for health systems.

Lastly, further advancements in imaging technology and innovations such as AI should help to further enhance COPD diagnosis, disease characterization, and risk assessments [259,260]. More routine utilization of objective criteria to define phenotypes, endotypes, etiotypes, and severity of COPD exacerbations [84,85,261] would be helpful to more precisely study risks, outcomes, and optimal interventions. Better disease characterization should enhance future personalization of therapies. This could include treatment of pre-COPD, PRISM, and COPD at an earlier stage in the course of disease—before advanced and irreversible structural changes in the lungs and/or comorbidities are present.

A better understanding of the cellular and molecular pathogenesis of COPD should help to guide the development of a broader array of options for biologic and other therapies targeted to specific patient subgroups based on identified disease mechanisms and pathways [232,238,239,247,262]. Promising areas of active investigation include treatments targeting alarmins (such as TSLP and IL-33), non-Th2 inflammatory pathways, kinases, phosphodiesterases, oxidative stress, or the cystic fibrosis transmembrane regulator and agents to modify the pulmonary microbiome [232,247]. These approaches should help guide optimal use of existing biologic therapies. For example, given that high blood eosinophil count predicts lung function decline [263], might biologic therapies targeting Th2 inflammation slow lung function decline? Might biologic therapies also improve outcomes for people with Th2 inflammation even without frequent exacerbations? Is there a role for biologic therapies for people with COPD in the peri-exacerbation period [262]?

Other important areas to explore include prevention of small airway loss, methods for slowing alveolar destruction and/or enhancing alveolar restoration [264,265,266], and understanding the effects of biologic therapies on systemic manifestations and comorbidities (such as vasculature, cardiac function, systemic inflammation, etc.).

Further work is also needed to understand the impact of novel weight loss medications (such as GLP-1 receptor agonists and SGLT2 inhibitors) on the pulmonary and systemic manifestations and comorbidities of COPD; information to date suggests reductions in the risk of COPD exacerbations among obese adults with diabetes and reductions in obstructive sleep apnea [267,268,269,270,271].

In summary, future treatments for COPD will hopefully be both more personalized based on knowledge of the disease mechanisms and more focused on disease modification. This should help to reduce the burden of COPD on patients, as well as the impact of COPD on health systems.

## 13. Limitations

While this narrative review attempts to capture many of the important themes in our evolving understanding of COPD, it is important to note that it reflects expert perspectives, is not a comprehensive review, and focuses mainly on Western history. We hope that we presented many of the significant advances that have been made in this field, but we recognize that much more work is needed, especially in the areas of neutrophilic inflammation, the cellular and molecular pathogenesis of COPD, and effective recognition and treatment of early stage disease. We look forward to the work of future expert clinicians and scientists to further our understanding of this complex and fascinating disease.

## Figures and Tables

**Table 1:** A non-exhaustive list of selected interventions targeting hyperinflation in patients with COPD.

Interventions	Effects on Hyperinflation
Pharmacological	
Inhaled bronchodilators	Larger effects on static and dynamic hyperinflation compared to FEV_1_ Dual bronchodilator therapy (LABA + LAMA) is superior to monotherapy Lung deflation on exercise was closely related to improved neuromechanical dissociation and dyspnea, even in a subset of symptomatic patients with preserved FEV_1_ and mild air trapping
Inhaled steroids	Added positive effect to long-acting bronchodilators by mildly reducing static and dynamic hyperinflation
Roflumilast	Mild but statistically significant increase in exercise IC with no change in “static” hyperinflation
Opiates	Inhaled fentanyl was associated with mild improvement in exercise IC and exertional dyspnea
Inhaled furosemide	Improvement in exercise IC and lower operating lung volumes in some dyspneic individuals
Non-pharmacological	
Exercise training	Less dynamic hyperinflation by decreasing ventilatory requirements and respiratory rate; these effects were seen even in patients with severe “static” hyperinflation Greater beneficial effects on hyperinflation seen with interval versus constant load training
Oxygen	By decreasing the ventilatory demands, it may contribute to lessening dynamic hyperinflation
Heliox	Variable improvement in dynamic hyperinflation; potential adjunct effect of helium-hyperoxia
Inspiratory muscle training	May enhance patients’ ability to deal with exercise-induced dynamic hyperinflation
CPAP	May counterbalance hyperinflation-induced PEEPi, improving exertional dyspnea
BiPAP	Variable positive effects on exercise IC, particularly in patients with more severe static hyperinflation
LVRS	Beneficial consequences to dynamic hyperinflation and, consequently, diaphragm shape and excursion
Bronchoscopic lung reduction	Decreased static and dynamic hyperinflation, notably in those with higher air trapping

**Table 2:** Domains that influence PR referral and participation. Modified with permission from Cox et al. [213].

Theoretical Domain	Barriers	
**Knowledge**	HCPs-how to refer HCPs- who to refer Patients-why they were referred Patients-not heard of PR	
**Environmental Context**	Waiting list Burden of referral Burden of CRD Burden of co-existing conditions	Travel Time requirements Cost Baseline characteristics
**Beliefs About Consequences**	Lack of perceived benefit Disease too severe or not severe enough Fear of breathlessness Fear of exacerbation of other conditions Beliefs about role of exercise Beliefs about safety of exercise	
**Social Influences**	Social or family support (living alone) Attitude of HCP Negative impact of the group setting	

CRD = chronic respiratory disease.

**Table 3:** Summary of PR recommendations. Modified from Rochester et al. [217].

Question	Recommendation	Strength of Recommendation Quality of Evidence
1. Should adults with stable COPD undertake PR?	For adults with stable COPD, we recommend participation in pulmonary rehabilitation	Strong Moderate
2. Should adults with COPD undertake PR following hospitalization for AECOPD?	For adults with COPD, we recommend participation in pulmonary rehabilitation following hospitalization for an exacerbation	Strong Moderate
3. Should adults with ILD undertake PR?	For adults with ILD, we recommend participation in pulmonary rehabilitation.	Strong Moderate
4. Should adults with pulmonary hypertension undertake PR?	For adults with pulmonary hypertension, we suggest participation in pulmonary rehabilitation	Conditional Low
5. Should adults with chronic respiratory disease undertake tele-rehabilitation?	For adults with stable chronic respiratory disease, we recommend offering the choice of centre-based PR or tele-rehabilitation	Strong Moderate
6. Should adults with chronic respiratory disease undertake maintenance PR?	For adults with COPD, we suggest either supervised maintenance pulmonary rehabilitation or usual care after initial pulmonary rehabilitation	Conditional Low

COPD = chronic obstructive pulmonary disease; ILD = interstitial lung disease.

## Data Availability

No new data were created or analyzed in this study. Data sharing is not applicable to this article.
